# Oxytocin Involvement in Body Composition Unveils the True Identity of Oxytocin

**DOI:** 10.3390/ijms22126383

**Published:** 2021-06-15

**Authors:** Claudia Camerino

**Affiliations:** 1 Department of Biomedical Sciences and Human Oncology (Section of Pharmacology), School of Medicine, University of Bari Aldo Moro, P.za G. Cesare 11, 70100 Bari, Italy; ccamerino@libero.it; 2 Department of Physiology and Pharmacology “V. Erspamer”, Sapienza University of Rome, P.le Aldo Moro 5, 00185 Rome, Italy

**Keywords:** oxytocin, thermoregulation, skeletal muscle, obesity, Prader–Willi syndrome

## Abstract

The origin of the Oxytocin/Vasopressin system dates back about 600 million years. Oxytocin (Oxt) together with Vasopressin (VP) regulate a diversity of physiological functions that are important for osmoregulation, reproduction, metabolism, and social behavior. Oxt/VP-like peptides have been identified in several invertebrate species and they are functionally related across the entire animal kingdom. Functional conservation enables future exploitation of invertebrate models to study Oxt’s functions not related to pregnancy and the basic mechanisms of central Oxt/VP signaling. Specifically, Oxt is well known for its effects on uteri contractility and milk ejection as well as on metabolism and energy homeostasis. Moreover, the striking evidence that Oxt is linked to energy regulation is that Oxt- and Oxytocin receptor (Oxtr)-deficient mice show late onset obesity. Interestingly Oxt^−/−^ or Oxtr^−/−^ mice develop weight gain without increasing food intake, suggesting that a lack of Oxt reduce metabolic rate. Oxt is expressed in a diversity of skeletal muscle phenotypes and regulates thermogenesis and bone mass. Oxt may increases skeletal muscle tonicity and/or increases body temperature. In this review, the author compared the three most recent theories on the effects of Oxt on body composition.

## 1. Introduction

Peptides are employed as messenger molecules in different communication systems involved in the control of physiological processes and behavior [1]. These systems that include wiring transmission (communication via the synapses), volume transmission (communication via the intercellular space), hormonal transmission (communication via the circulation), and environmental transmission, emerged during various stages of evolution [2]. Complex organisms such as vertebrates recruited many peptides and receptors into the hormonal communication channel, thus increasing the demand for specificity in order to exclude cross-activation of other receptors. Oxytocin (Oxt) and Vasopressin (VP) evolved from a single ancestral gene and have acquired distinct regions of many amino acid residues that exclude cross-activation receptors [3]. Multiple receptors may have existed before separate lineages of VP/Oxt-related peptides evolved. The evolution of functionally distinct peptides lineages, from a common ligand–receptor pair, requires not only the specificity on both the peptides and the receptors but also a differential cellular pattern of expression of distinct receptor types. This suggests that the existence of differentially expressed receptor subtypes was important in the functional divergence of VP and Oxt [2]. Nevertheless, a certain degree of pleiotropy is conserved in several peptides including Oxt. The importance of structural characterization of VP and Oxt was recognized in the award of the 1955 Nobel Prize for Chemistry to Vincent de Vigneaud [4]. This program of research started in 1895 when Oliver and Schafer reported that a substance extracted from the pituitary gland elevates blood pressure when injected intravenously into dogs [5]. This vasopressor originates from the neurohypophysis [6] while it was later reported that a neurophysical substance triggers uterine contractions [7,8] together with other bioactivities of pituitary extracts like stimulation of lactation and antidiuresis [9]. Purification of pituitary gland extracts revealed that the vasopressor and antidiuretic effect could be attributed to one substance (VP) and the uterotonic and lactation effect could be attributed to another substance (Oxt) [10]. In the 1950s, the amino acid sequences and secondary structure of VP [11,12] and Oxt [13,14] were determined and both peptides were chemically synthesized [14]. This revealed that VP (CYFQNCPRG-NH2) and Oxt (CYIQNCPLG-NH2) differ by only two amino acids indicating a common evolutionary origin back millions of years ago, which shows also that Oxt has effects that go beyond pregnancy. About 10 years ago or so, our laboratory raised the notion that Oxt regulates energy and metabolism. Indeed, mice that are homozygous for deletions of either Oxt or its receptor develop late onset obesity and metabolic syndrome. Interestingly, Oxt- and Oxtr-deficient mice develop a metabolic phenotype in the absence of hyperphagia [15,16]. The metabolic role of Oxt diverges in young versus older animals or, alternatively, it takes time to reach full force. This concept was named in our laboratory as “The oxytocin paradox”. This discrepancy can be explained by the fact that Oxt may only mark the identity of neurons projecting from Paraventricular nucleus of the hypothalamus (PVN) but its action is mediated by classical neurotransmitters like GABA, alternatively, Oxt may be anorexigenic in normal mice, but compensatory mechanisms may take place in Oxt^−/−^ or Oxtr^−/−^ mice [17,18,19]. Another hypothesis was that the appetite of Oxt^−/−^ reported as normal, in spite of the hyperleptinemia, was excessive relative to the degree of adiposity [20]. This hypothesis was ruled out by the evidence that the stomachs of Oxt^−/−^ mice were comparable to wild-type mice for size and weight excluding any excess in food consumption [16]. A very important point is that Oxtr-deficient mice have a basal temperature lower than wild-type, which shed a light on the role of Oxt on temperature regulation and lean/fat mass composition in this model [15]. Oxt signaling is important for whole-body glucose metabolism and Oxt administration in humans reducing fasting respiratory quotient meaning that Oxt can induce a shift from carbohydrate to fat oxidation [21,22]. Recently, and about 10 years after these first discoveries, three different studies have been published by Yuan et al. [23], Sun et al. [24], Conte et al. [19], each of them presenting a different theory on the role of Oxt in the regulation of energy metabolism and the paradox of normophagic obesity that we first described for Oxt^−/−^ and Oxtr^−/−^ mice [15,16]. Specifically, in Yuan et al. [23], the author analyzed the role of Oxt in energy homeostasis after a cold stress (CS) challenge and found that Oxt dissipates stored energy in brown adipose tissue (BAT) and white adipose tissue (WAT) improving metabolic dysfunctions in obese mice. Moreover, Oxt induces precursor adipocytes differentiation towards brown adipocytes after CS consistent with the needs of non-shivering thermogenesis. The exposure of mice to CS increased Oxt expression in hypothalamus, in BAT and iWAT (inguinal WAT) as well as circulating Oxt. Importantly CS increases Oxt expression in skeletal muscle since thermogenesis in skeletal muscle plays an important role for whole body energy. In the Yuan et al. [23] study, Oxt infusion has no effect on the reduction of food consumption in diet induced obese mice. The author explained this data set postulating that Oxt regulates thermogenesis. This may bring one to the conclusion that the normophagic obesity of Oxt^−/−^ and Oxtr^−/−^ mice was caused by dysfunctional regulation of body temperature since they found that Oxt infusion increases body temperature. On another note, Sun et al. [24] showed that Oxt stimulates the synthesis of new bone which can be useful during pregnancy and lactation when fetal demands of calcium are high although this effect cannot be attributed only to Oxt since this hormone increases only during the very last part of pregnancy and for a very limited time. Moreover, they found that Oxt reduces body fat and reduces white to brown transition, probably as a compensatory mechanism to conserve energy after the loss of body weight. Lastly, in the study of Conte et al. [19], the author analyzed the involvement of Oxtr/Transient-receptor-potential-vanilloid-1 (TRPV1) genes in the adaptation of skeletal muscle to CS in mice and found that Oxtr increases in hypothalamus. Circulating Oxt decreases after CS following a negative feedback loop in the brain. Oxtr increases in skeletal muscle is phenotype dependent with Oxt potentiating the slow-twitch muscle phenotype through the regulation of Myosin heavy chain 1 (slow oxidative)/Myosin heavy chain 2 b (fast glycolytic) ratio after CS consistent with the shivering needs of thermogenesis. Oxt increases in bone after CS to balance the decreases of circulating Oxt. The author concludes that Oxt increases skeletal muscle tonicity in the same manner it does with the uteri triggering what they called “The oxytonic contractions” after CS and they attribute the normophagic obesity of Oxt^−/−^ and Oxtr^−/−^ to a general loss of skeletal muscle tonicity rather than to the increase in food consumption.

In this review, we described and compared these three theories by Yuan et al. [23], Sun et al. [24], and Conte et al. [19] on the role of Oxt in body composition and outside of pregnancy with the intent of incorporating them into one complete theory.

## 2. Oxytocin Functions Outside of Pregnancy: A Step Back, Oxytocin–Vasopressin System in Invertebrates

Phylogenetic analysis reveals that neuropeptide signaling systems are evolutionarily ancient [25] and the evolutionary origin of at least 30 neuropeptide families including VP/Oxt-type was found back to the bilaterian common ancestor of the deuterostomes that includes vertebrate and invertebrate chordates, hemichordates, and echinoderms and prostasomes that includes arthropods, nematodes, mollusks, and annelids [26,27,28]. VP/Oxt-related peptide was found in protostomia (most invertebrates) and deuterostomia (some invertebrates and all vertebrates) lineages [29,30]. More recently, comparative analysis of genome–transcriptome sequence data revealed the discovery of genes encoding VP/Oxt-type neuropeptides in species belonging to different phyla [31,32]. Nevertheless, the discontinuous conservation of VP/Oxt-related peptides in several species is caused by the adaptation to different lifestyles [31]. In vertebrates, four types of VP/Oxt receptors are classified with distinct expression patterns and biological effects. The VP/Oxt receptor family is most closely related to the superfamily of gonadotropin-releasing hormone receptors, which have also been found in a wide variety of deuterostomia and protostomian lineages [33,34]. Investigations of the actions of VP/Oxt-type neuropeptides in chordates, and protostomian invertebrates has revealed conserved roles in the regulation of processes such as reproduction and water homeostasis [31,35]. Pleiotropic effects of the VP/Oxt family in homeostatic regulation include the control of stress responses, metabolism and circadian rhythms. VP and Oxt are myoactive peptides that stimulate contractions in a variety of tissues. Myoactivity is one of the best conserved functions of VP and Oxt-related peptides [30,36]. Interestingly this has provided evidence that not only the structures but also the functions of VP/Oxt- type neuropeptides are evolutionarily conserved. Thus, a VP/Oxt-type neuropeptide in insects named Isotocin has a VP-like role in regulating urine production [37], whereas a VP/Oxt-type neuropeptide in the mollusk Lymnaea stagnalis has an Oxt-like role in regulating reproductive physiology [38,39]. Comparative investigation of the physiological roles of VP/Oxt-type neuropeptides has largely focused on vertebrates and selected protostomian invertebrates. Echinotocin homolog of Oxt has been identified in the sea urchin Strongylocentrotus purpuratus and in vitro studies revealed that echinotocin acts as a muscle contracting in sea urchins, consistent with the myostimulatory actions of VP and Oxt in mammals [30]. Echinotocin causes contraction of in vitro preparations of the esophagus and tube feet in sea urchins [30]. This effect of echinotocin as a muscle contractant is consistent with the myoexcitatory effects of VP/Oxt type neuropeptides in many other taxa and with our theory [19,40]. In vertebrates, VP/Oxt-type neuropeptides are mostly known for their effects on reproductive physiology/behavior, feeding and digestive process and osmoregulation [41,42] and Oxt inhibits feeding [43,44] in mammals. Similarly, also in the bilaterian annelid Eisenia Foetida, the VP/Oxt-type neuropeptide annetocin potentiates spontaneous contractions of gut preparations, providing evidence of a role in the regulation of digestive processes and tonicity of the involuntary muscle [19,45]. Annetocin also reduces the body weight of leeches [46] and evokes contractions of the earthworm’s excretory nephridia [36] although these effects are more likely to be interpreted as reproduction-related actions [47,48]. In the cephalopod mollusk Octopus vulgaris, the VP/Oxt-type signaling system has been characterized in detail and Octopus vulgaris is the only invertebrate that comprises two VP-Oxt-type neuropeptides, octopressin (OP), and cephalotocin (CT) and three cognate receptors (OPR, CTR1, and CTR2). Furthermore, OP is expressed in the regions of the octopus’s nervous system, involved in the control of feeding and/or gut activity and accordingly OP has myoexcitatory effects on in vitro preparations of the octopus’s rectum and cardiovascular tissues expressing the octopressin receptor [49,50,51]. However, experimental evidence that OP regulates feeding behavior in an octopus has not yet been reported. Interestingly, extensive analysis in the nematode *C. elegans* has revealed that, in addition to the reproductive role in the regulation of mating behavior [52], VP/Oxt-type signaling regulates gustatory associative learning in these species [53]. At a molecular level, nematocin, analog of Oxt signaling in nematodes, acts in a genetic pathway for gustatory plasticity interacting at a molecular level with TRPV1 [53,54] consistent with Oxt activation of TRPV1 in skeletal muscle of mice [19]. Thus, both direct and indirect evidences that VP/Oxt-type signaling is involved in the regulation of feeding-related processes have been obtained from a variety of studies on protostomes. In this phylogenetic survey, the only invertebrate in which VP/Oxt-neuropeptide function is myorelaxant rather than myoexcitatory is the starfish where the homolog asterotocin has relaxing effect on muscle preparations. Investigation of the in vitro pharmacological actions of the VP/Oxt-type neuropeptide asterotocin in the starfish A. Rubens, revealed an unusual characteristic since it acts as a muscle relaxant, whereas in other taxa, VP/Oxt-type neuropeptides typically cause muscle contraction [5,38,52,55]. Asterotocin is released physiologically by neurons located in the mucosal layer of the cardiac stomach and then diffuse in the visceral muscle layer to act on asterotocin receptors on muscle cells causing relaxation. Injections of asterotocin induces fictive feeding in starfish triggering both cardiac stomach eversion and adoption of a body posture resembling that which occurs during natural feeding on prey. This is why asterotocin has a physiological role as a neural regulator of this unusual extra-oral feeding behavior of starfish [25]. Asterotocin triggers fictive feeding in starfish reflecting an evolutionary ancient role of VP/Oxt-type neuropeptides as regulators of feeding related process in the bilateria. This adds to the existing evidence of evolutionary ancient roles in the regulation of diuresis, reproductive processes, and reflects the pleiotropy of neuropeptides functions in animals [25]. In conclusion, invertebrate studies underscore the conservation of hormonal effects of VP/Oxt-system on peripheral target tissues including the control of myoactivity and reproduction [30,36,39]. In this context, it is reasonable to hypothesize that from the moment animals started exploring new environments where they encountered a variety of novel cues related to mating partners or food availability the emergence of a neuropetidergic system that could accordingly direct their behavior and decisions was a great benefit to their survival [31].

## 3. Yuan et al.’s Theory and Oxytocin Regulation of Thermogenesis in Skeletal Muscle

The aim of this study was to treat precursors adipocytes and obese mice with Oxt. The author found that Oxt induces brown adipocytes formation and stimulates thermogenesis in BAT, iWAT, and skeletal muscle. The points of thermogenesis and skeletal muscle as Oxt’s target organ are very important and we found them also in Conte et al.’s theory [19] as we will see below. The role of Oxt in energy homeostasis was examined and found that Oxt dissipates stored energy in WAT and BAT improving metabolic dysfunction in obese mice. Oxt induces precursor adipocytes differentiation towards brown adipocyte. This is another important point since Sun et al. [24] states the opposite. In this study, Yuan et al. used a cold stress (CS) protocol that includes an acclimation period of 1 h under 6 °C at day 1 and 2 h under 6 °C at day 2 and finally 4 h under 6 °C for 6 days. Starting day 4 mice were kept at 6 °C for 8 days while control mice were kept at 20–24 °C. After CS treatment mice were sacrificed under anesthesia and tissues collected for RNA while serum for Oxt measurement. The results of this work can be summarized in three major steps: (1) Mice were exposed to CS and found that Oxt expression was increased in hypothalamus. (2) Circulating concentration of Oxt increases as well because Oxt can cross the blood–brain barrier (BBB) from brain to periphery [56] but not periphery to brain [57]. This secretion of Oxt probably comes from the central neural system since magnocellular neurons of hypothalamus is a predominant site for Oxt production. (3) CS induces Oxtr expression in multiple peripheral tissues including adipose tissue. Indeed Oxt in plasma is elevated and Oxtr in iWAT and BAT was also enhanced suggesting these tissues are targeted by Oxt to produce heat for cold acclimation. UCP1 in BAT was also increased. In this study, CS induces Oxt mRNA expression in hypothalamus and Oxtr in adipose tissue. In high fat diet induced obese mice Oxt delivered by mini pumps increased body core temperature decreasing body weight gain. Oxt induced thermogenic genes expression in BAT, WAT, and skeletal muscle. In summary, Oxt induced iWAT browning by inducing PRDM16 that is a potent activator of brown adipocyte formation [58] in C3H10T1/2 cell line, Oxt stimulates thermogenesis in BAT, WAT, and skeletal muscle promoting thermogenesis and combating obesity. WAT is energy storage and BAT is energy dissipation to heat production. Beige adipose tissue is discovered in WAT, contains UCP1 and after adrenergic stimulation or CS beige is induced in BAT with similar functions in heat production [59,60]. Beige adipose tissue is promising to combat obesity and Oxt is a novel therapeutic agent to combat obesity [61,62,63,64]. Previous studies show that deletion of Oxt/Oxtr induces late on-set obesity in mice [15,16] and Oxt administration reduces body weight among different species [65,66,67]. Downstream of these results Yuan et al. [23] formulates three possible hypothesis to explain this mechanism. The first explanation seems to be reduced food intake. However, unaltered food consumption was observed in Oxt/Oxtr^−/−^ mice [15,16] and is consistent with Yuan et al. [23] that showed that Oxt infusion did not affect food intake in high fat diet induced obese mice. Food intake was also not altered by Oxt in diet induced obese rats or leptin receptor deficient db/db [68,69]. Nevertheless, these results could also be caused by difference in treatment routine compared to other studies. The second explanation for reduced body weight gain induced by Oxt is increased energy expenditure. Oxt infusion in the third ventricle stimulates energy expenditure and lipolysis [67,69,70]. However, exogenous Oxt application does not affect locomotor activity meaning that there are other potential factors contributing to enhanced energy expenditure [71,72,73]. The third explanation is that Oxt-mediated body weight loss may be caused by increased thermogenesis since heat production related thermogenesis could be another way to regulate energy expenditure [74]. Oxt administration induced thermogenesis in BAT and lipolysis in WAT by increasing sympathetic nervous system activity [64]. Interacting with Oxtr, Oxt produces lipolysis in adipocytes [67] and in Oxt^−/−^ mice sympathetic tone is decreased which can cause late onset obesity [16]. This indicates that Oxt is a potent inducer of thermoregulation and two pathways are responsible for this action, a direct pathway with Oxt interacting with Oxtr or indirect pathway activating sympathetic nervous system activity. In Yuan et al. [23] experiments this probably happened via a direct pathway since serum adrenaline was not altered in cold stressed mice while Oxt infusion increased body temperature and promoted UCP1 expression in iWAT and BAT. Mice with Oxt neuron ablation have lower BAT temperature when exposed to 4 °C for three hours [75]. Yuan et al. [23] found that in addition to adipose tissue also in skeletal muscle genes involved in heat production, fat acid transport and lipolysis were stimulated by Oxt. This means that thermogenesis in skeletal muscle may also be activated by Oxt by direct pathways since skeletal muscle express Oxtr [76]. The author concluded that thermogenesis in skeletal muscle plays an important role for whole body energy like adipocytes. This is a very important point also found in Conte et al. [19]. Chronic administration of Oxt induces continuous decrease in body weight gain also after cessation probably for Oxt self-stimulatory properties [66,67,72]. Beige fat becoming brown are a drug target for developing agents against obesity and associated disorders. PRDM16 is a transcriptional factor for determining adipocytes differentiation towards beige adipocytes [58]. In this study, Oxt promotes brown adipocytes specific gene expressions in differentiated C3H10T1/2 cells, and PRDM16 plays an important role in mediating such effects. Oxt induces a decrease in white adipocytes selective markers that maybe due to activation of PRDM16 by Oxt that repress white adipocyte selective gene expression in favor of brown adipocyte. In sum: Oxt acts as a white adipose tissue browning inducer. The author concludes that Oxt supplement can be useful for dealing with obesity and metabolic syndrome. Nevertheless, Oxt has side effects like uterine contractions. During labor circulating Oxt increases by seven fold and Oxtr in myometrium by 150 fold [77,78]. In clinical applications, the induction of Oxt mRNA during parturition requires the decline in progesterone and the incline in estrogens levels after luteolysis [79]. This is why the current doses of Oxt to treat obesity cannot elicit contractions.

## 4. Sun et al.’s Theory and Oxytocin Regulation of Bone Mass and Brown Fat

The aim of this study was: (1) to explore the effect of deleting Oxtr specifically on osteoblasts (OB) and osteoclasts (OC) using transgenic mice expressing Cre driven by 2.3-kb Colla1 or Acp5 promoter the gene encoding type 5A tartrate-resistant acid phosphatase (TRAP), respectively, and (2) to analyze the effect of Oxt infusion on body weight, and found that Oxt inhibits feeding through a central action on PVN. The rational of this work is that Oxt is a bone anabolic hormone that promotes OB maturation to a mineralizing phenotype and regulates genesis and function of bone resorbing OC [80,81,82]. Nevertheless, it has been reported that central Oxt has a negative effect on bone mass and the lack of Oxt leads to high bone mass as a secondary effect of hyperleptinemia [83]. The physiological explanation for this mechanism could be the intergenerational transfer of calcium ions during pregnancy and lactation in favor of the formation of the fetus skeleton and for lactation. Oxt is a potent regulator of bone mass through its direct action on Oxtr on OB and OC [80,81,82]. Global deletion of Oxt/Oxtr results in profound age associated osteopenia [82]. Oxt stimulates OB to a more mineralizing phenotype while it has a dual action on OC. Oxt enhances OC formation from hematopoietic stem cells precursors but inhibits the activity of mature OC by inhibition of nitric oxide [82,84]. It remains unclear at the light of a reduced bone mass in Oxt^−/−^ and Oxtr^−/−^ if OC action dominates. In this work, Sun et al. [24] state that in humans’ and rodents’ plasma Oxt rise in late pregnancy and lactation a period coinciding with demineralization of the maternal skeleton for the fetal skeleton. The maternal skeleton is then repaired without loss of bone with excessive bone loss leading to osteoporosis of pregnancy and lactation [85]. However, Oxt rises only during the very last part of pregnancy and for a short period of time just enough to elicits uterine contractions and when the fetus skeleton is already formed [79]. The work of Sun et al. [24] can be divided in two separate segments. In the first segment, Sun et al. analyzed the effect of Oxt on OB and OC generating a tissue specific KO. Sun et al. deleted Oxtr in OB and OC using Col2.3Cre and Acp5Cre mice, respectively. Regarding Oxt action on OB, Sun et al. [24] found that both male and female Col2.3Cre^+^:Oxtr^fl/fl^ recapitulate the low bone mass phenotype of Oxtr^+/−^ suggesting that Oxt has a prominent osteoblastic action in vivo. Moreover, abolishment of the anabolic effect of estrogens in Col2.3Cre^+^:Oxtr^fl/fl^ mice suggests that osteoblastic Oxtr_s_ are necessary for estrogens action. Regarding the OC study, Sun et al. [24] find that the high bone mass in Acp5Cre:Oxtr^fl/fl^ mice indicates a prominent action of Oxt in stimulating osteoclastogenesis. Oxt stimulates the genesis of OC while inhibiting the resorptive activity of mature cells. Which one of these actions, stimulation of OC genesis or inhibition of resorption, contributes to the skeletal action of Oxt? In this regard, Sun et al. [24] formulated the “brake theory” meaning that under calcium stress Oxt mobilize calcium but can also be used as a “brake” preventing excessive bone dissolution. The high bone mass phenotype in Oxtr-less OCs can be attributed to reduction of osteoclastogenesis because Oxt favors the formation of new osteoclasts, but Oxt also prevents unrestricted resorption putting a brake on their activity. Moreover, the actions of pharmacologic doses of 17β-estradiol used in postmenopausal women for osteoporosis appears to be mediated by Oxt. Estrogens stimulate the expression of Oxt and Oxtr in OB [80,86,87]. In this work, Sun et al. [24] hypothesized that the lack of Oxtr in OB of Col2.3Cre^+^Oxtr^fl/fl^ mice abolishes estrogens anabolic action on the skeleton. This is in contrast to the non-requirement of Oxt for hypogonadal bone loss in ovariectomized (Ovx) mice and in Oxtr^−/−^ [88], but is consistent with high FSH and low estrogens that contribute to bone loss post Ovx and that is rescued after estrogens replacement and administration of anti-FSH antibodies [89,90]. Their dataset established Oxt signaling as necessary for the anabolic action of estrogens on bone and confirm no function of Oxt in bone loss of hypogonadism. Nevertheless, hypogonadal Oxt^−/−^ mice present high bone mass consequent to hyperleptinemia [83,91]. In the second segment of their research Sun et al. [24] focused on the effects of Oxt on metabolic functions. Oxt may in addition to the effects on bone mass also regulates body composition. High levels of Oxtr mRNA and proteins were found in WAT and lower in BAT. The application of Oxt to adipocyte derived from 3T3L1 precursors inhibits the beiging gene program. Oxt may interacts with AVpr1a, that is a VP receptor, on adipocytes although Oxt does not interact with AVpr1a on OBs [89]. Sun et al. [24] confirmed the existence of Oxtr on adipocytes by Western and Sanger sequencing. Further experiments are needed to delete Oxtr in adipocytes. Oxt triggered 17β-hydroxysteroids dehydrogenase which convert estrone to estradiol in adipocytes. In this regard, Sun et al. [24] hypothesized that high Oxt in pregnancy may stimulate estrogen production from WAT to compensate for pregnancy associated lowering of estrogens [88]. In clinical application, the induction of Oxt during parturition requires the decline in progesterone and the incline in estrogens levels. This is why this theory is not applicable since an increase in estrogens during pregnancy would probably correct osteoporosis but induce preterm birth [79]. Sun et al. [24] find that Oxt acts on Oxtrs in adipocytes to suppress white to beige transition gene program. Despite this direct antibeiging action, injected Oxt reduces total body fat. This finding is in contrast with the Yuan et al. study [23] that found Oxt having beiging actions on WAT. Sun et al. [24] explains that the antibeiging action of Oxt in their experiment is probably to save energy during Oxt-induced weight loss. However, WAT is energy storing and BAT is energy dissipation, this is why physiologically it is more plausible that Oxt induces WAT beiging to promote non-shivering thermogenesis after weight loss. It is important to underline that the Sun et al. [24] experiments were not performed after CS exposure that is a physiological activator of BAT and beige adipose tissue. Moreover Oxt stimulates Serpine1 gene that encodes for plasminogen activator inhibitor 1 (PAI1). This may contribute to the anti-thrombotic state in pregnancy when Oxt is high. The action of Oxt used to prevent excessive post-partum bleeding may arise from PAI1 expression. In sum, Oxt regulates bone mass, body composition, metabolism, and thrombosis during states of physiological stress when Oxt is high, like in pregnancy and lactation. The late-onset obesity of Oxtr^−/−^ [15], is normophagic; however, both subcutaneous and intraperitoneal Oxt injections modify food intake. this is why Sun et al. [24] suggest that Oxt cross the BBB although many evidences state the opposite [57]. Indeed, central Oxt administration with minimal spillover to the periphery increases insulin secretion from β-cell via the vagal cholinergic system, whereas peripheral Oxt in the circulation induces glucagon to a greater extent relative to insulin. This is probably caused by a direct hepatic and α-cell interaction, leading to increased hepatic glucose output. The reduction of caloric intake by suppression of Hypothalamic-Pituitary-Adrenal axis activity is one of the mechanism by which Oxt improves postprandial metabolic parameters [92]. However, Oxt^−/−^ and not only Oxtr^−/−^ as cited by Sun et al. [24] present late on set obesity and unaltered food consumption [16] but Sun et al. [24] did not mention this study [16] in their paper. This work describes a new effect of Oxt through activation of Oxtr_s_ on adipocyte and a cell autonomous antibeiging action of Oxt to conserve energy that compensate the centrally mediated reduction in body fat. In conclusion, Sun et al. [24] showed that Oxt regulates bone mass and body fat. Oxt stimulates the synthesis of new bone while preventing bone loss during pregnancy and lactation, Oxt reduces body fat but as a compensatory mechanism prevents white to beige transition to conserve energy. Sun et al. [24] also agree that Oxt/Oxtr are targets for future therapies for osteoporosis and obesity or for diseases as Prader–Willi syndrome or obese patients with SIM1 gene mutation that presents reduced numbers and size of Oxt-ergic neurons in PVN [93,94].

## 5. Conte et al.’s Theory and Oxytocin Driven Increased Tonicity of Skeletal Muscle and the “Oxytonic Effect”

The aim of this study was to explore the involvement of Oxtr/TRPV1 genes and Oxt on the adaptation of skeletal muscle to CS in mice. Oxt/Oxtr mRNA was measured in Soleus (Sol) and Tibialis anterioris (TA) by RT-PCR. Oxtr expression was analyzed in PVN and Supraoptical nucleus (SON) and hippocampus (HIPP) by immunohistochemistry and circulating Oxt was measured in plasma. Potentiation of slow-twitch muscle after CS is observed in rat and mice [95,96]. Oxt may lead to the activation of transmembrane ion channels permeable to calcium ions like the TRPV1 cation channel which plays a key role as a thermal and analgesic effector in different tissues [97]. TRPV1 mediates the pain signaling of Oxt in neurons and Oxt other than Oxtr may directly interact with TRPV1 as previously seen for Oxt analogue in invertebrates [53,54,98,99]. However, Oxt expression in different muscle phenotypes after CS and Oxt driven increase in muscle tone necessary for shivering thermogenesis has not yet been investigated. Recently, it has been shown that Oxt/Oxtr are implicated in the regulation of energy homeostasis [15,16]. Oxt/Oxtr^−/−^ mice show late onset obesity but are normophagic and this is probably caused by reduced metabolic rate and energy expenditure [16]. The lack of Oxtr leads to impaired thermoregulation and decreased core body temperature after acute exposure to cold [15,100,101]. Skeletal muscle is also a source of heat in CS animals and humans through voluntary contractions from exercising muscle or involuntary as contractions from shivering muscle [102]. CS activates the involuntary activation of skeletal muscle movements. ATP is necessary to sustain muscle contraction [102,103]. Oxtr is present in human myoblasts and Oxtr agonists increases the rate of myoblasts fusion and myotubes in the culture [56,81,99,104,105]. Oxt is a paracrine/autocrine agent that regulates the tonicity of human skeletal muscle as seen in invertebrates [31]. Oxt was first described for its tonic smooth muscle regulation of gastric motility showing that exogenous Oxt excited circular muscle strips and isolated smooth muscle of the gastric body [106]. Oxt contracts the slow-twitch muscle of mammary gland and myometrium [107]. Based on this rational in an interorgan approach to the physiology of CS, Conte et al. [19] formulated the hypothesis that Oxt may contract all the slow-twitch muscles as Oxt contracts the utero having a tonic, thermogenic and analgesic effect. The hypothesis that Oxt is implicated in thermogenesis through skeletal muscle activation is consistent with Yuan et al. theory [23]. Conte et al. [19] generated the paradigm shifting hypothesis that the metabolic syndrome of Oxt/Oxtr^−/−^ mice was caused by muscular failure and depotentiation being Oxt a slow-twitch muscle enhancer. Yuan et al. [23] explained the normophagic obesity of Oxt/Oxtr^−/−^ mice with the implication of Oxt in thermoregulation also in skeletal muscle. Oxt improves glucose uptake in C2C12 myoblasts by stimulation of intracellular calcium release and activation of AMP-activated protein kinase. Oxt treatment downregulates the expression of fatty acid binding protein 4, a protein associated with reduced skeletal muscle growth and trans-differentiation of myotubes and muscle-derived stem cell into adipocyte-like cells. Oxt may also directly influence glucose metabolism through promotion of muscle cell differentiation and proliferation via extracellular signal-regulated kinase phosphorylation in the mitogen-activated protein kinase pathways. Oxtr binding in human myoblast cultures was shown to promote myoblast fusion. However, the main peripheral effects of Oxt are located in adipose tissue rather than skeletal muscle as the expression of Oxtr is higher in adipose tissue than skeletal muscle in lean rats and expression levels in WAT were comparable to classical Oxt target tissues [92]. Nevertheless, the expression level of Oxtr in skeletal muscle increases after thermogenic stress and is phenotype dependent [19,24] This is consistent with the increase of myosin heavy chain 1 (slow-oxidative)/myosin heavy chain 2b (fast-glycolytic) (Mhc1/Mhc2b) gene expression ratio in Sol but not in TA muscle together with the upregulation of the Oxtr gene in Sol muscle [40]. Brain Oxt may up-regulate the short-term response of Sol, while it may down-regulate the brain–Sol intercommunication after long-term exposure to CS as shown by a linear correlation curve in a feed-forward/feed-back regulation between the brain and Sol [40,96,108]. This means that low circulating Oxt levels are required for a better response to long-term CS challenge. Nevertheless, the Oxt signaling is maintained by the up-regulation of Oxtr gene found in Sol muscle after long term CS that balances the reduced level of circulating Oxt consistent with previous studies [80]. Conte et al. [19] found that Oxt expression in hypothalamus and Oxtr in adipose tissue were induced by CS consistent with Yuan et al. data [23] regulating both shivering and non-shivering thermogenesis. Conte et al. shows [19] by immunohistochemistry that CS increases Oxtr in PVN and Hipp after both long and short term CS exposure consistent with gene expression data in whole brain [108]. A different pattern of Oxtr is observed in SON, the major site of Oxt secretion where Oxt is unchanged at 6 hours and decreased at 5 days. Oxtr and TRPV1 genes increased after 6 hours and 5 days CS in Sol and TA. Oxtr/TRPV1 genes expression in Sol and TA evaluated by regression analysis was linearly correlated after CS but not at thermoneutrality consistent with the coupling between these two genes at CS. The circulating levels of Oxt are unaffected after 6 hours, but decreased after 5 days. This data is consistent with data from the correlation curve [40], but is in contrast with the Yuan et al. data that find circulating Oxt decreasing after CS [23]. The actions of Oxt can be mediated by Oxtr that is a type A of GPCR responsible for the release of calcium from the intracellular stores and activation of PKC. The TRPV1 cation channel is a thermal and analgesic effector in different tissues. TRPV1 mediates the pain signaling of Oxt in neurons. Circulating Oxt other than Oxtr can directly interact with TRPV1 [97,98,99]. This is consistent with the hypothesis that Oxt has analgesic effects. In this work, Conte et al. [19] showed that CS induces the expression of TRPV1 and Oxtr in skeletal muscle and is higher in slow-twitch skeletal muscle. Circulating Oxt leads to activation of Oxtr and TRPV1 channel on membrane. Oxtr gene expression increased at 6 hours and 5 days CS in Sol and TA. TRPV1 increased after 6 h and 5 days CS in Sol and TA. Regression analysis shows a significant linear correlation between Oxtr and TRPV1 in Sol and with a lesser extent in TA. The correlation Oxtr and TRPV1 in Sol and TA was lost at thermoneutrality. The hearts of CS mice were also examined. Cardiac muscle is an Oxt target organ and Oxt is cardioprotective and prevent fibrosis, hypertrophy, and inflammation [76,109]. Db/db mice are obese with reduced Oxtr in heart [68]. Oxt treatment prevents cardiomyopathy of db/db independently of hyperphagia and hyperleptinemia caused by a direct effect on cardiac muscle [68]. This is consistent with the hypothesis of a direct effect of Oxt on skeletal muscle and the heart. The heart is a target organ for Oxt and expresses Oxtr. Conte et al. [19] speculated that Oxt protects the cardiac muscle from necrotic process and increases its tonicity as shown by histological studies and by the fact that mice rescued their body weight after CS treatment which is a sign of good health. In this work, Conte et al. [19] showed that PVN presents reduced gene expression and density of Oxtr after CS and low circulating Oxt [110,111]. Prader–Willi Sindrome patients (PWS) and Nectin-deficient mice, a Prader–Willi syndrome model present impaired thermogenesis, metabolic syndrome, reduction of Oxt producing neurons in PVN while circulating Oxt is high [94,112]. This phenotype is the striking mirror image of CS mice in Conte et al. study [19]. PWS patients are hypotonic at birth and this hypotonic state proceeds in late onset obesity because PWS has a defect in Oxt transmission [110,113]. However, to confirm these preliminary findings a study of Oxt/Oxtr in different skeletal muscle phenotypes of PWS would be necessary. In sum: a reduced Oxtr in PVN leads to increased Oxt secretion by the posterior pituitary due to the loss of negative feedback. The low temperature increases Oxtr expression in PVN and in Sol followed by the decreases in circulating Oxt. This mechanism was named by Conte et al. [19] as the “The Oxytonic effect” that is mediated by Oxtr and TRPV1 in skeletal muscle and increases the tonicity of the slow-twitch muscle and has analgesic effects. PWS has high circulating Oxt. This is in contrast to the expectation that hypothalamic Oxt decreases food intake by increasing leptin concentration in plasma. This may be caused by a sort of “Oxt resistance” as previously seen for leptin [114] or alternatively for high levels of inactive forms and not enough of the active forms. Alternatively, Oxtr may be suppressed after genetic or metilation defect. Oxt has beneficial “Oxytonic effect” in skeletal muscle through adaptation of Oxtr/TRPV1 after long term CS with analgesic effects and increasing its tonicity. The up-regulation of Oxtr in PVN balances the decrease in circulating Oxt. Moreover, Leptin and Oxt present close interactions that are important for the regulation of food intake and body weight homeostasis. Treating high fat diet fed hyperleptinemic mice with Oxt re-established a normal anorexigenic effect and body weight after acute but not chronic treatment with leptin. The beneficial Oxt effects after acute treatment with leptin is that Oxt and leptin would act synergistically to activate the Central Nervous System centers involved in the control of food intake. Oxt-induced body weight loss is mainly food intake independent as also chronic Oxt treatment decreased body weight without long-term modification of food intake [115]. Moreover, Oxt increases fat oxidation, improves insulin sensitivity and glucose transport in peripheral tissues of mice nevertheless marked species differences in the expression patterns of Oxtr suggests that some of its observed effects may be species-specific. The effects of Oxt differ also in obese and diabetic state. Intranasal administration of Oxt in human subjects was associated with significant weight loss and improvements in insulin sensitivity, pancreatic function and lipid homeostasis that strongly suggest a role for this system as a therapeutic target in obesity and diabetes. The complexity of obesity and the pathogenesis of obesity-related metabolic complications underscore the need to better understand the role of Oxt in metabolic functions [92]. This novel pathway that we described in skeletal muscle may help diseases characterized by obesity and in PWS.

## 6. Conclusions

In the three theories described above, the authors agree that Oxt has important functions outside of pregnancy that include the regulation of energy homeostasis and bone metabolism and that understanding these functions can be useful to treat diseases like obesity and metabolic syndrome or Prader–Willi syndrome that is closely correlated with the Oxt system.

**Figure 1:**
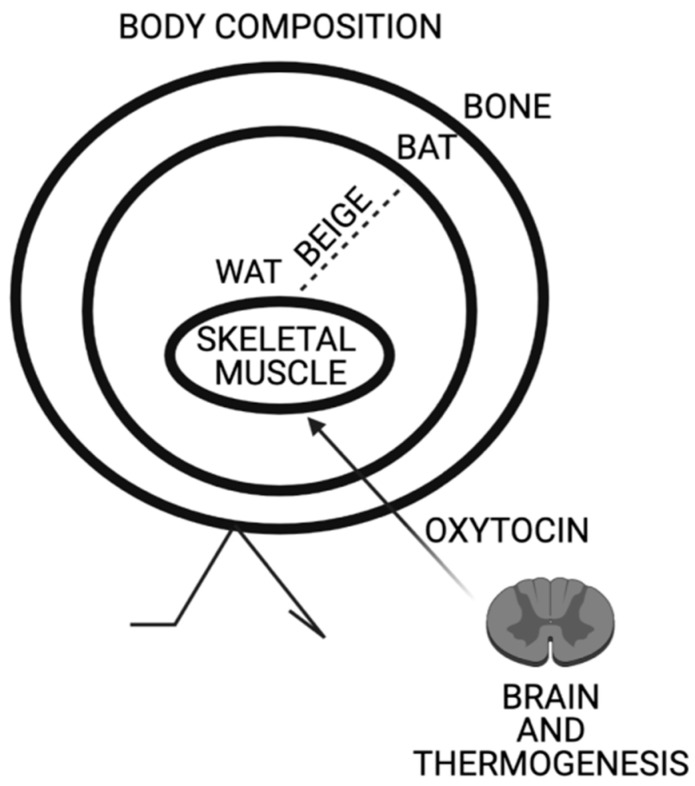
Schematic representation of Oxytocin effects on body composition. Oxytocin expression in paraventricular nucleus is triggered by thermogenesis. Oxytocin acts on skeletal muscle increasing its tonicity and thermogenic genes. Oxytocin induces browning of white adipose tissue and peripheral Oxytocin increases bone mass.

Specifically, Oxt regulates thermoregulation in both shivering and non-shivering fashion through the regulation of thermogenic genes in skeletal muscle and browning of WAT (Figure 1). The myoexitatory effect of Oxt in parallel with the activation of TRPV1 is conserved through evolution for over 600 million years as shown by its presence in several invertebrates and mollusks. The functions of Oxt are much wider than uteri contractility and the true physiological identity of Oxt appears to be linked to the regulation of energy metabolism through the functionality of skeletal muscle. The author hopes that this review contributes to highlight a new and non-unitarian perspective of Oxt physiology.

## Abbreviations

BAT	Brown adipose tissue
WAT	White adipose tissue
iWAT	Inguinal adipose tissue
CS	Cold stress
CSF	Cerebrospinal fluid
BBB	Blood brain barrier
HIPP	Hippocampus
MyHC	Myosin heavy chain
Oxt	Oxytocin
Oxtr	Oxytocin receptor
PVN	Paraventricular nucleus
PWS	Prader–Willi syndrome
SON	Supraoptical nucleus
Sol	Soleus muscle
TA	Tibialis anterioris
TRPV1	Transient receptor potential vanilloid 1
PAI1	Plasminogen activator inhibitor 1
AVpr1a	Vasopressin receptor
